# Using an Advanced Practice Pharmacist in a Team-Based Care Model to Decrease Time to Hemoglobin A_1c_ Goal Among Patients With Type 2 Diabetes, Florida, 2017–2019

**DOI:** 10.5888/pcd17.190377

**Published:** 2020-06-04

**Authors:** Kevin Cowart, Wendy Updike, Nnadozie Emechebe, Janice Zgibor

**Affiliations:** 1 Department of Pharmacotherapeutics & Clinical Research, Taneja College of Pharmacy, Morsani College of Medicine, University of South Florida, Tampa, Florida; 2 Department of Internal Medicine, Morsani College of Medicine, University of South Florida, Tampa, Florida; 3 Department of Family Medicine, Morsani College of Medicine, University of South Florida, Tampa, Florida; 4 Department of Epidemiology, College of Public Health, University of South Florida, Tampa, Florida

## Abstract

Collaborative practice models that use an advanced practice pharmacist (APP) have been shown to improve outcomes for patients with chronic diseases. Few studies have evaluated the effects of team-based practice models involving an APP for time needed to attain glycated hemoglobin A_1c_ (HbA_1c_) goals in patients with diabetes mellitus (type 2 diabetes). Ours is a retrospective cohort study, involving patients with type 2 diabetes who worked with a pharmacist in an academic family medicine clinic. These patients experienced a shorter time to achieve an HbA_1c_ of less than 7%, as compared with patients who did not work with a pharmacist. Future studies should evaluate the length of time patients can sustain an HbA_1c_ of less than 7% with team-based care involving an APP and the influence of such care on diabetes-related complications.

**Summary**

**What is already known on this topic?**

Few studies have evaluated the influence of team-based practice models involving an advanced practice pharmacist (APP) on the time needed to reach a hemoglobin A_1c_ goal. APPs function in a way similar to a mid-level provider, adjusting antidiabetic medications and providing diabetes self-management education under a defined scope of practice.

**What is added by this report?**

As compared with usual medical care, a team-based practice model using an APP led to a shorter median time to reach a hemoglobin A_1c_ goal of less than 7% in patients with type 2 diabetes mellitus.

**What are the implications for public health practice?**

Team-based care involving an APP might lead to improvements in glycemic control, which have the potential to decrease the burden of diabetes as a chronic disease.

## Objective

Achievement of treatment goals for patients with type 2 diabetes is suboptimal. Only half of the patients with diabetes achieve a glycated hemoglobin A_1c_ (HbA_1c_) of less than 7% (1), despite the availability of effective antidiabetic therapy and clinical practice guidelines that are updated annually (2). Timely achievement of an HbA_1c_ goal might have a beneficial effect on clinical outcomes, such as development of macrovascular and microvascular complications of type 2 diabetes (3). The aim of our study was to analyze the time to achieve an HbA_1c_ of less than 7% for a pharmacist–physician managed (PPM) cohort, as compared with a usual medical care (UMC) cohort of patients with type 2 diabetes.

## Methods

Our retrospective cohort study was conducted between January 2017 and July 2019, at the University of South Florida (USF) Health Morsani Center, Department of Family Medicine. Inclusion criteria were adults, aged 18 to 80 years, having type 2 diabetes for least 12 months, and an HbA_1c_ at 7% or higher at the index visit (the first visit during the study). Inclusion criteria were confirmed by chart review. Demographic and clinical data were collected from existing medical records. Exclusion criteria were confirmed pregnancy (because gestational diabetes typically involves more stringent glucose control) or a documented endocrinology visit during the study (to evaluate influence of the nonbiased, team-based practice model). Our study was certified exempt by the USF Institutional Review Board.

We assigned patients to the PPM cohort if they had at least 1 visit with their primary care physician (PCP) in the past 3 years and at least 1 visit with an advanced practice pharmacist (APP) in the USF Health Department of Family Medicine. Patients were assigned to the UMC cohort if they were managed solely by their PCPs and did not have a clinic visit with an APP during the study. A collaborative drug therapy management agreement gave APPs the authority to initiate, titrate, or discontinue antidiabetic medications; order drug therapy–related laboratory tests; and provide diabetes self-management education. APPs practiced in the same clinic as the PCP and independently saw patients face-to-face following referrals from the PCP. Visits with the APP were scheduled for 30 to 60 minutes, whereas visits with patients in the UMC cohort were 20 to 40 minutes. Visits in the UMC cohort were either routine follow-up visits or sick visits.

Median time (in days) to an HbA_1c_ less than 7% was calculated by using the Kaplan-Meier estimate among each cohort and compared by using a log rank test. To account for the PPM cohort having increased interactions with patients, each cohort was categorized into 3 groups, based on adherence to their visit schedules. Adherence to visit schedules was calculated as a proportion by dividing the total number of actual interactions with a PCP, an APP, or both by the total number of expected interactions for each patient (sum of actual, no-show, and canceled visits). Therefore, if a participant in the PPM cohort had 12 PCP and 8 APP visits during the study without any missed visits, then the patient would have an adherence value of 1 (20 visits / 20 expected interactions = 1). Similarly, if a patient in the UMC cohort had 12 visits with the PCP without any missed visits, then the patient was assigned an adherence value of 1. This approach, rather than the actual number of interactions, accounts for the disparate number of expected interactions inherent with the PPM cohort relative to the UMC cohort. Patients were subsequently categorized as having low, moderate, and high visit adherence according to the distribution of the adherence scores. The entire patient population was stratified by adherence and the study groups (PPM vs UMC) were compared within each stratum. The analysis was also stratified by the median HbA_1c_ value.

Chi-square tests and 2 independent sample *t* tests were used to determine whether a statistically significant difference existed between groups for categorical and continuous variables, respectively, at baseline. Follow-up time was calculated as time in days from first visit to achieving the HbA_1c_ goal or last clinic visit. Statistical significance was defined as *P* < .05. The analysis was conducted using SAS version 9.4 (SAS Institute, Inc).

## Results

A total of 257 patients were included (n = 76 in the PPM cohort and n = 181 in the UMC cohort) with a median follow-up time of 357 days (interquartile range, 199–538 days). Groups did not differ substantially at baseline, except for HbA_1c_, which was significantly higher in the PPM cohort as compared with the UMC cohort (*P* < .001). For characteristics of the study population, mean age was 59.4 (SD = 11.8) years, 63.2% were white, and mean duration of type 2 diabetes was 3.2 (SD = 1.2) years. Mean body mass index was 34.2 kg/m^2^ (SD = 7.7). More than half (56.7%) of patients were commercially insured and 41.6% of patients were former or current tobacco smokers (Table 1).

**Table 1:** Participants with Type 2 Diabetes by Adherence Group^a^, Florida, 2017–2019

Characteristic	All Participants (N = 257)	Adherence in PPM Cohort			Adherence in UMC Cohort			*P* Value
		Low (n = 24)	Moderate (n = 30)	High (n = 22)	Low (n = 62)	Moderate (n = 54)	High (n = 5)	
**Age, mean (SD), y**	59.4 (11.8)	56.4 (12.0)	62.1 (9.9)	60.2 (12.3)	57.7 (12.9)	57.7 (12.3)	61.9 (10.5)	.13
**Race, n (%)^b^**								
Non-Hispanic black	69 (27.6)	11 (45.8)	7 (23.3)	9 (40.9)	19 (32.2)	15 (28.8)	8 (12.7)	<.001
Non-Hispanic white	158 (63.2)	13 (54.2)	21 (70.0)	11 (50.0)	34 (57.6)	27 (51.9)	52 (82.5)	
Other	23 (9.2)	0	2 (6.7)	2 (9.1)	6 (10.2)	10 (19.2)	3 (4.8)	
**Insurance, n (%)^b^**								
Commercial	139 (56.7)	12 (54.5)	12 (42.9)	12 (57.1)	33 (55.0)	34 (65.4)	36 (58.1)	.74
Government^c^	104 (42.4)	10 (45.5)	16 (57.1)	9 (42.9)	27 (45.0)	17 (32.7)	25 (40.3)	
Medicaid	2 (0.8)	0	0	0	0	1 (1.9)	1 (1.6)	
**Smoking status, n (%)**								
Never smoked	150 (58.4)	13 (54.2)	17 (56.7)	15 (68.2)	34 (54.8)	32 (59.3)	39 (60.0)	.71
Quit smoking	81 (31.5)	6 (25.0)	11 (36.7)	6 (27.3)	19 (30.6)	18 (33.3)	21 (32.3)	
Currently smoke	26 (10.1)	5 (20.8)	2 (6.7)	1 (4.5)	9 (14.5)	4 (7.4)	5 (7.7)	
**BMI, mean (SD)**	34.2 (7.7)	34.7 (8.1)	36.1 (9.5)	35.9 (8.9)	34.0 (6.3)	34.2 (8.0)	32.9 (7.1)	.49
**Duration of diabetes, mean (SD), y^d^**	3.2 (1.2)	3.2 (1.4)	3.4 (1.5)	3.1 (1.1)	3.1 (1.2)	3.0 (1.1)	3.2 (1.1)	.76
**Baseline HbA_1c_, mean (SD), %**	8.5 (1.6)	9.4 (1.9)	9.2 (1.7)	9.0 (1.8)	8.4 (1.4)	8.1 (1.4)	8.2 (1.4)	<.001
**Days to HbA_1c_, mean (SD)**	371 (206)	376 (188)	412 (200)	353 (221)	345 (197)	371 (198)	383 (226)	.77
**HbA_1c_ goal met**								
No	141 (54.9)	16 (66.7)	11 (36.7)	11 (50.0)	38 (61.3)	29 (53.7)	36 (55.4)	.25
Yes	116 (45.1)	8 (33.3)	19 (63.3)	11 (50.0)	24 (38.7)	25 (46.3)	29 (44.6)	
**Clinic visits**								
Actual visits, mean (SD)	7.8 (4.9)	9.2 (4.4)	13.7 (5.1)	13.0 (7.3)	5.4 (2.6)	6.5 (3.4)	6.2 (2.5)	<.001
Canceled visits, mean (SD)	3.2 (3.2)	8.3 (5.8)	4.7 (1.9)	1.8 (1.7)	4.2 (2.5)	2.4 (1.3)	0.7 (0.7)	<.001
No-shows, mean (SD)	0.5 (1.0)	1.4 (1.9)	0.7 (1.3)	0.3 (0.6)	0.8 (1.0)	0.4 (0.7)	0.0 (0.2)	<.001
Actual visits: expected visits, mean (SD)^e^, %	70.7 (17.3)	50.0 (9.6)	72.5 (4.4)	88.4 (7.3)	52.2 (8.8)	69.8 (4.2)	89.9 (8.2)	<.001

Abbreviations: BMI, body mass index; HbA_1c,_ glycated hemoglobin A_1c_; PPM, pharmacist–physician managed; SD, standard deviation; UMC, usual medical care.

a Adherence calculated as a proportion by dividing the total number of actual interactions with a primary care physician, advanced practice pharmacist, or both by the total number of expected visits for each patient.

b Percentages are based on available data from the electronic health record. Not all data were available.

c Government represents benefits from Civilian Health and Medical Program of the Department of Veterans Affairs, Medicare, or Medicare Advantage.

d Duration defined as number of years since diabetes or prediabetes was diagnosed.

e Expected visits are the sum of actual visits, no-shows, and canceled visits.

Median time to achieve an HbA_1c_ of less than 7% in the PPM cohort was 470 days, as compared with 569 days in the UMC cohort (median difference of 99 days, *P* = .60) (Table 2). However, when results were stratified by baseline HbA_1c_, the median time to achieve an HbA_1c_ of less than 7% was 512 and 668 days for the PPM and UMC cohorts, respectively (*P* = .11). Similarly, when results were stratified by adherence to clinic visits, the median time to achieve an HbA_1c_ of less than 7% in the PPM cohort was 441 days based on moderate adherence to clinic visits, and 381 days based on high adherence to clinic visits (Table 2). Among those included in the PPM cohort with low adherence to clinic visits, time to achieve an HbA_1c_ less than 7% was not estimable. That is, 50% of patients in the PPM cohort with low adherence to clinic visits did not achieve an HbA_1c_ less than 7%, based on the time specified in this analysis. Among patients in the UMC cohort, time to achieve an HbA_1c_ less than 7% was 612 days for those with low adherence, 457 days for moderate adherence, and 569 days for high adherence. However, these differences were not statistically significant (*P* = .80).

**Table 2:** Analysis of Glycated Hemoglobin A_1c_ Goal Achievement Between Pharmacist‑Physician Managed Care and Usual Medical Care, Florida, 2017–2019

Goal Achievement	Pharmacist–Physician Managed		Usual Medical Care		*P* Value
	Patients Who Met HbA_1c_ Goal, No./Total	Time to HbA_1c_ Goal, Median (95% CI), d	Patients Who Met HbA_1c_ Goal, No./Total	Time to HbA_1c_ Goal, Median (95% CI), d	
**Overall**	38/76	470 (372.0–NE)	78/181	569 (437–707)	.60
**Cohort classification by baseline HbA_1c_**					.11
Less than median (<8%)	12/20	380 (224.0–NE)	57/104	437 (383.0–638.0)	NA
Greater than or equal to median (≥8%)	26/56	512 (372.0–NE)	21/77	668 (612.0–NE)	NA
**Stratification by adherence to visit schedule**					.80
Low adherence	8/24	NE	24/62	612 (424.0–NE)	NA
Moderate adherence	19/30	441 (335.0–NE)	25/54	457 (392.0–NE)	NA
High adherence	11/22	381 (263.0–NE)	29/65	569 (383.0–867.0)	NA

Abbreviations: CI, confidence interval; HbA_1c_, glycated hemoglobin A_1c_; NA, not applicable; NE, not estimable.

In the PPM cohort, 50% of patients met an HbA_1c_ goal of less than 7%, as compared with 43.1% in the UMC cohort (*P* = .31). When stratified by adherence to clinic visits, 33.3% of patients in the PPM cohort with low adherence to clinic visits met an HbA_1c_ goal of less than 7%, as compared with 38.7% of patients with low adherence to clinic visits in the UMC cohort. A higher percentage of patients in the PPM cohort with moderate adherence (63.3%) and high adherence (50.0%) to clinic visits met an HbA_1c_ goal of less than 7% compared with patients in the UMC cohort with moderate and high adherence to clinic visits (46.6% and 44.6%, respectively).

## Discussion

Patients exposed to an APP in our study (PPM) experienced a shorter median time to achieve an HbA_1c_ of less than 7% than did those receiving usual care (UMC), although results were not statistically significant. To account for an expected greater number of clinic visits in a team-based care practice model using an APP, results were stratified by adherence to clinic visits. The stratified analysis demonstrated that patients in the PPM cohort with moderate and high adherence to clinic visits had a shorter median time to reach an HbA_1c_ of less than 7% compared with the UMC cohort with the same level of clinic visit adherence. Among patients who had a high level of adherence to clinic visits, the mean and median time to an HbA_1c_ of less than 7% was shorter in the PPM cohort than in the UMC cohort. These results are not surprising, especially in light of additional time spent with the APP for diabetes self-management education. Our findings also highlight the need for new methods to improve adherence and outreach, especially among patients with low to moderate adherence to clinic visits.

Our findings confirm the results in 2 similarly designed studies (4,5). In the first, time to HbA_1c_ goal was 23 days shorter among patients exposed to a collaborative practice model involving an APP, as compared with usual care (4). Among those with a baseline HbA_1c_ more than 8% and exposed to an APP, the time to HbA_1c_ goal was 144 days shorter (*P* < .05), as compared with usual medical care (4). In a second study, time to HbA_1c_ goal was 125 days shorter in patients exposed to an APP, as compared with usual care, although results were not statistically significant (5). However, the clinical significance of these findings is meaningful because a shorter duration of uncontrolled diabetes might decrease the risk of developing microvascular and macrovascular complications (6).

Our study has limitations. First, because the study design is retrospective, information obtained is dependent on existing documentation in the medical record. Second, because patients in the PPM model were also managed by their PCP, attributing positive outcomes to the PPM intervention alone is a challenge. The same limitation might apply to the UMC cohort, as PCPs might often consult with the APP informally for drug selection recommendations for patients who might not have been included in the PPM cohort.

Third, information about use of antidiabetic medication during the study, which might influence HbA_1c_, was not collected. Finally, patients may have been referred to the APP for poorly controlled diabetes (as indicated by higher baseline HbA_1c_ in the PPM cohort), creating the potential for bias from inclusion in the PPM cohort. Findings indicate that implementation of a team-based practice model involving a pharmacist in an academic family medicine setting might shorten time to achieve the HbA_1c_ goal, although cautious interpretation with respect to level of adherence to clinic visits is needed. Additional research needs to include confirmation of our findings in a larger sample size, evaluation of medication-related treatment intensification, and qualitative barriers to treatment intensification.
